# Abscopal effect in metastatic breast cancer treated with stereotactic body radiotherapy in the absence of immunotherapy

**DOI:** 10.3389/fonc.2023.1243053

**Published:** 2023-10-06

**Authors:** Jae Sik Kim, Ah Ram Chang

**Affiliations:** Department of Radiation Oncology, Soonchunhyang University Seoul Hospital, Soonchunhyang University College of Medicine, Seoul, Republic of Korea

**Keywords:** abscopal effect, breast cancer, metastasis, neutrophil-to-lymphocyte ratio, stereotactic body radiotherapy

## Abstract

**Purpose:**

In this study, we aimed to assess the abscopal effect (AE) after CyberKnife stereotactic body radiotherapy (SBRT) in metastatic breast cancer patients without immunotherapy.

**Methods:**

We reviewed breast cancer patients who received SBRT with a fraction size of ≥ 6 Gy for metastatic lesions between July 2008 and December 2021. We selected patients who had at least one measurable extracranial lesion in addition to SBRT target lesions and were not treated with immunotherapy. A total of 40 SBRT cases from 34 patients were included in the analysis. The AE was defined as occurring before the overall progression of the disease, regardless of the use of systemic treatment.

**Results:**

The median follow-up duration was 16.4 months. Among 40 SBRT cases, the AE was observed in 10 (25.0%) with a median interval of 2.1 months. Of these lesions, 70.0% did not progress for one year. In multivariate logistic regression analysis, no change in systemic treatment after SBRT was significantly associated with an increase in the AE (odds ratio [OR] = 1.428, 95% confidence interval [CI] = 1.108 – 1.841, p = 0.009). A post-SBRT neutrophil-to-lymphocyte ratio (NLR) of < 2 marginally increased the AE (OR = 1.275, 95% CI = 0.998 – 1.629, p = 0.060). However, a high SBRT dose and large planning target volume did not (p = 0.858 and 0.152, respectively) in univariate analysis.

**Conclusions:**

One out of four patients experienced the AE after SBRT in the absence of immunotherapy. The AE could occur more frequently when systemic treatment remains unchanged, and patients have a low NLR after SBRT.

## 1 Introduction

The abscopal effect (AE) was first described in 1953 and referred to an interesting systemic anticancer response following localized radiotherapy (RT), resulting in the regression of other tumorous lesions not targeted by RT (1, 2). Although the underlying mechanism of the AE has not been well defined, several hypotheses have been suggested, including neoantigen presentation, chemokine release, immune cell activation, and the reduction of immunosuppressive factors, all of which are induced by RT (3). The AE is rarely observed, but as a potential therapeutic approach, radiation oncology societies have shown significant interest in it, particularly with the rise of immunotherapy (4).

A shorter treatment duration with a larger fraction size leads to the activation of the type-I interferon pathway, which is correlated with the AE (5). Stereotactic body radiotherapy (SBRT), which delivers high radiation doses in fewer fractions with high precision, is the most effective treatment in terms of the AE. The increased expression of *DNASE1* following SBRT was associated with cytolytic T-cell gene expression and tumor-promoting cells, such as M2 macrophages, were less stimulated by SBRT (3, 6).

The AE can be further boosted by combining SBRT with immunotherapy (3). A systematic review demonstrated that the weighted mean of the AE using SBRT and immunotherapy was 41.3% (range, 26.0 – 67.0), better than that of immunotherapy alone (weighted mean, 17.7%) (7). To enhance the AE, the addition of low-dose radiotherapy (LDRT), having no cytotoxic effect, to SBRT has been attempted (8, 9). MD Anderson Cancer Center conducted a non-randomized phase II trial and reported the significantly improved lesion-specific response of LDRT lesions compared to non-irradiated lesions (9). In this trial, immunotherapy continued.

A retrospective analysis of a phase II study showed that the AE was observed in 45% of patients receiving SBRT-based partial tumor irradiation targeting the hypoxic segment (SBRT-PATHY) for unresectable bulky non-small cell lung cancer in the absence of immunotherapy (10). This treatment showed higher immunogenic potential due to the sparing of the peritumoral immune-microenvironment (PIM), where locoregional immune cells can be activated by massive tumor neoantigens released after SBRT-PATHY (4). However, there may be a limitation to applying SBRT-PATHY, as the tumor should be bulky with sufficient hypoxic regions.

At our institution, we performed SBRT using CyberKnife and assumed that the steep dose fall-off of the CyberKnife would allow for the relative sparing of the PIM. Additionally, we aimed to investigate the role of LDRT in the AE in patients with metastatic breast cancer who did not receive immunotherapy. Therefore, we evaluated the AE observed after CyberKnife-based SBRT in metastatic breast cancer patients and explored potential predictors affecting the AE and survival outcomes.

## 2 Materials and methods

This single-institution retrospective study was approved by the Institutional Review Board at Soonchunhyang University Seoul Hospital (No. SCHUH 2022-07-019). The requirement for informed consent was waived due to the retrospective review and minimal risk to patients. The study was conducted in accordance with the Declaration of Helsinki.

The study retrospectively reviewed patients who received SBRT for metastatic breast cancer lesions between July 2008 and December 2021 using CyberKnife. Patients with at least one measurable extracranial lesion in addition to SBRT targets were included. The fraction size of SBRT had to be equal to or more than 6 Gy. Patients were included who were treated with systemic treatments except for immunotherapy before and/or after SBRT. The exclusion criteria were: (i) previous RT within one month before SBRT, (ii) incomplete SBRT, (iii) unavailable information on SBRT, and (iv) no follow-up imaging after SBRT. Finally, 40 cases of SBRT from 34 patients were analyzed.

Since SBRT was delivered in various dose-fractionation schemes, the equivalent dose in 2-Gy fractions with α/β = 10 (EQD2) was calculated. If patients received SBRT for ≥ 2 lesions with different regimens in the same period, the lowest EQD2 was used in the analysis.

All malignant lesions not targeted by SBRT in the recent computed tomography (CT) before SBRT were retrospectively reviewed. Bone metastases were excluded due to difficulty in measuring their extent on CT. Since three lesions were not discernible on non-contrast simulation CT scans or due to the presence of pleural effusion, they were excluded from the analysis. All 149 non-targeted lesions were divided into three groups: (i) not covered in simulation CT for SBRT (n = 62); (ii) outside the 0.5 Gy isodose line (n = 54); and (iii) inside the 0.5 Gy isodose line (n = 33). The mean EQD2 of non-targeted lesions inside the 0.5 Gy isodose line was measured.

The lesion-specific response was evaluated according to the Response Evaluation Criteria in Solid Tumours 1.1 after SBRT. The AE was defined as a complete or partial response of lesions that were not targeted by SBRT before overall disease progression, irrespective of the use of systemic therapies.

All statistical analyses were done with R statistical software, version 4.1.2 (https://www.r-project.org/). Box-and-whisker plots and mean dose profiles were generated by Prism 8.3.0 (GraphPad Software, San Diego, CA, USA). A p-value of less than 0.050 was considered statistically significant. Comparisons of categorical and continuous variables were conducted using Fisher’s exact test and the Mann-Whitney U test, respectively. The odds ratio (OR) for the AE was calculated using logistic regression models. Variables with statistical significance (p < 0.050) were used for multivariate analysis. Progression-free rate (PFR), progression-free survival (PFS) and overall survival (OS) were estimated by the Kaplan-Meier method, and the log-rank test was used to compare the survival outcomes between groups. Cox proportional hazards models were performed to identify the factors affecting PFS and OS. Per-treatment and per-lesion analyses were done for the AE, and PFS and OS were analyzed per treatment and per patient, respectively.

## 3 Results

The median follow-up duration for PFS and OS was 16.4 months (range, 3.2 – 157.7) and 27.4 months (range, 4.7 – 164.4), respectively. The baseline characteristics of 40 SBRT cases are summarized in **Table 1**. Of these, 18 (45.0%) patients had unresected primary breast cancer, and the median neutrophil-to-lymphocyte ratio (NLR) within one month after SBRT was 2.41 (range, 0.74 – 12.20). Bone was the most common site of SBRT (n = 29, 72.5%). The prevailing SBRT dose-fractionation scheme was 27 Gy in 3 fractions (n=18, 45.0%). An SBRT EQD2 of 42.75 Gy (range, 40 – 150) was delivered to the planning target volume (PTV) with a median volume of 40.81 cc (range, 2.53 – 129.74). After SBRT, systemic treatment was changed in 25 (62.5%) patients. In patients who did not have changes in systemic treatment, the second-line treatment had been used prior to SBRT, while in cases of changing in systemic treatment after SBRT, the patients had received the third-line treatment prior to SBRT on average.

**Table 1:** Baseline characteristics^*^.

Characteristics	N	% or range
Age, median (yr)	52.5	29.0-74.0
Molecular subtype		
HR+/HER2-	24	60.0
HR+/HER2+	8	20.0
HR-/HER2+	0	0.0
HR-/HER2-	8	20.0
Primary breast lesion		
Absent	22	55.0
Present	18	45.0
Post-treatment NLR, median	2.41	0.74-12.20
SBRT lesions^†^		
Bone	29	72.5
Liver	6	15.0
Lung	2	5.0
Others	4	10.0
SBRT regimen^‡^		
27 Gy in 3 fractions	18	45.0
30 Gy in 3 fractions	3	7.5
30 Gy in 5 fractions	5	12.5
33 Gy in 3 fractions	4	10.0
60 Gy in 3 fractions	4	10.0
Others	6	15.0
SBRT EQD2, median (Gy)	42.75	40.00-150.00
PTV, median (cc)	40.81	2.53-129.74
Systemic treatment before SBRT^†^	38	95.0
Chemotherapeutic agent	22	57.9
Targeted therapy	14	36.8
Hormone therapy	13	34.2
Systemic treatment after SBRT		
No change	14	35.0
Change	25	62.5
Stop	1	2.5

^*^Per-treatment analysis.

^†^Multiple.

^‡^If different dose-fractionation regimens were used during the same period, the regimen with the lowest equivalent dose in 2-Gy fractions (α/β = 10) was selected.

EQD2, equivalent dose in 2-Gy fractions (α/β = 10); HER2, human epidermal growth factor receptor 2; HR, hormone receptor; NLR, neutrophil-to-lymphocyte ratio; PTV, planning target volume; SBRT, stereotactic body radiotherapy.

**Figure 1:**
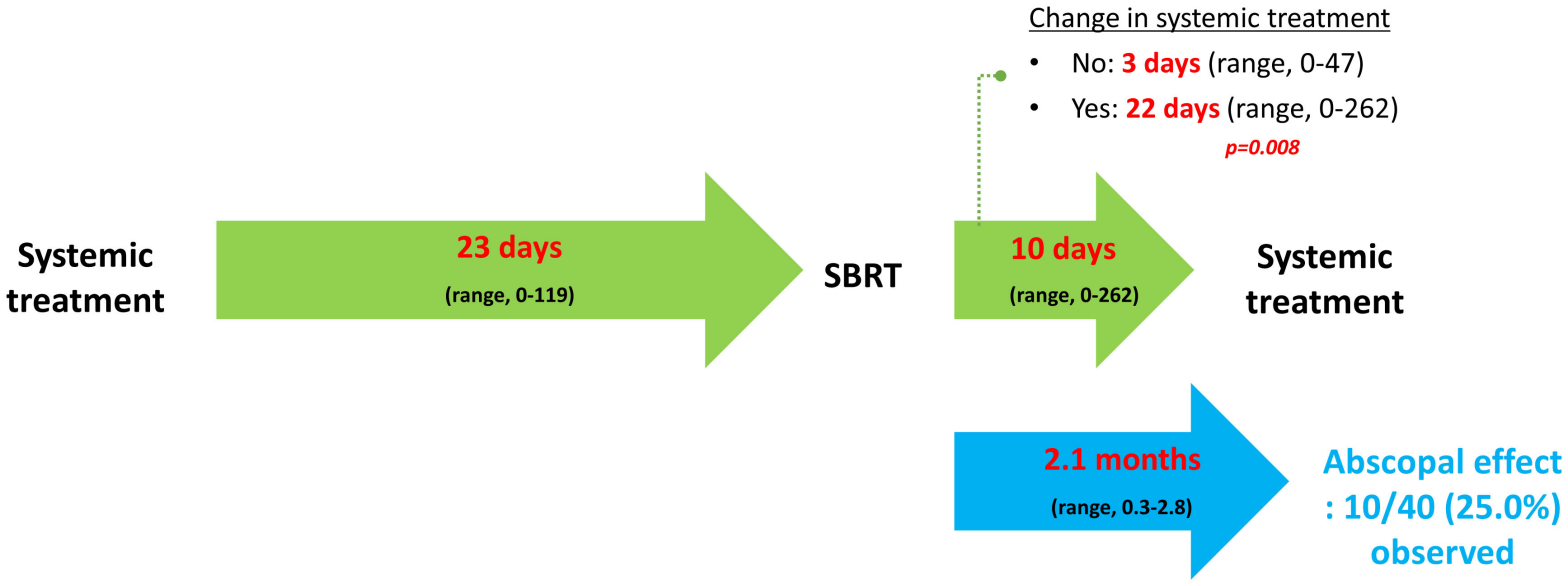
Temporal relationship between systemic treatment and stereotactic body radiotherapy. Per-treatment analysis. Median values are described with ranges. SBRT, stereotactic body radiotherapy.

**Figure 2:**
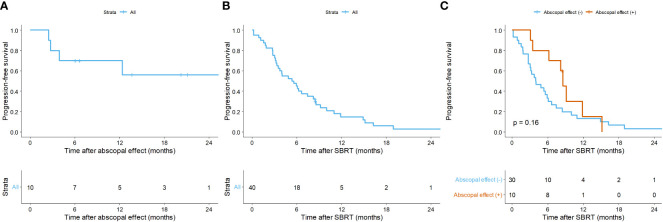
Kaplan-Meier curves of **(A)** progression-free rate of lesions with the abscopal effect, **(B)** progression-free survival (PFS) of overall lesions, and **(C)** PFS according to the abscopal effect. Per-treatment analysis. SBRT, stereotactic body radiotherapy.

**Figure 1** illustrates the time interval between systemic treatment and SBRT, and *vice versa*, described as median values with ranges. Notably, when physicians maintained the regimen, systemic treatment was immediately resumed (3 vs. 22 days, p = 0.008). The AE was observed in 10 (25.0%) cases at 2.1 months (range, 0.3 – 2.8) after SBRT. The 1-year PFR of lesions showing the AE was 70.0% (95% CI: 46.7 – 100.0; **Figure 2A**).

Among the 10 cases in the AE (+) group, systemic treatment was unchanged in seven (70.0%) cases, which was higher than that (n = 7, 23.3%) in the AE (–) group (p = 0.027; **Table S1**). Multivariate analysis showed that no change in systemic treatment after SBRT was significantly associated with an increase in the AE (OR = 1.428, 95% confidence interval [CI] = 1.108 – 1.841, p = 0.009; **Table 2**). Post-treatment NLR less than 2 marginally affected the AE (OR = 1.275, 95% CI = 0.998 – 1.629, p = 0.060). However, the timing of resuming systemic therapy after SBRT, as well as the SBRT dose and volume, were not prognostic factors of the AE.

**Table 2:** Analysis of the prognostic factors for the abscopal effect^*^.

	Univariate		Multivariate	
Variables	Odds ratio (95% CI)	P-value	Odds ratio (95% CI)	P-value
HR+/HER2- subtype	1.110 (0.840-1.467)	0.469		
Primary breast lesion present	1.164 (0.886-1.528)	0.283		
Chemotherapy before SBRT	0.788 (0.601-1.032)	0.092		
Targeted therapy before SBRT	1.210 (0.913-1.603)	0.193		
Hormone therapy before SBRT	1.252 (0.943-1.662)	0.129		
No change in systemic therapy after SBRT	1.469 (1.131-1.908)	0.006	1.428 (1.108-1.841)	0.009
Interval between SBRT and systemic therapy (incremental)	0.998 (0.996-1.000)	0.099		
Post-treatment NLR < 2	1.325 (1.018-1.725)	0.043	1.275 (0.998-1.629)	0.060
SBRT EQD2 > 42.75 Gy	0.975 (0.738-1.288)	0.858		
PTV > 40 cc	0.819 (0.626-1.070)	0.152		

^*^Per-treatment analysis.

CI, confidence interval; EQD2, equivalent dose in 2-Gy fractions (α/β = 10); HER2, human epidermal growth factor receptor 2; HR, hormone receptor; NLR, neutrophil-to-lymphocyte ratio; PTV, planning target volume; SBRT, stereotactic body radiotherapy.

**Figure 3:**
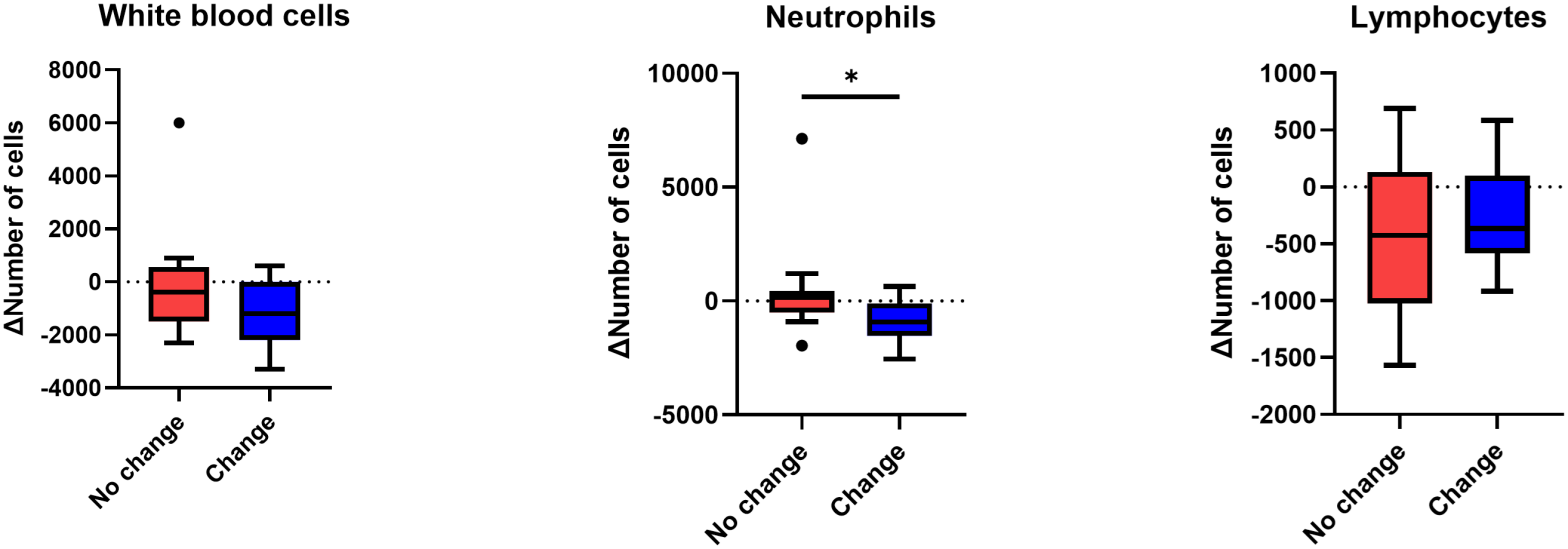
Change in the number of white blood cells, neutrophils, and lymphocytes after stereotactic body radiotherapy according to systemic regimen alteration. Per-treatment analysis. Only patients who had the results of complete blood cell counts within one month after radiotherapy, as well as after systemic treatment restarted following radiotherapy, were analyzed. Mann-Whitney U test was used, *p < 0.05.

In peripheral blood, a greater decrease in absolute neutrophil counts was observed when systemic treatment was changed (**Figure 3**). However, white blood cells (WBCs) and lymphocytes decreased similarly with or without systemic treatment alteration.

**Figure 4:**
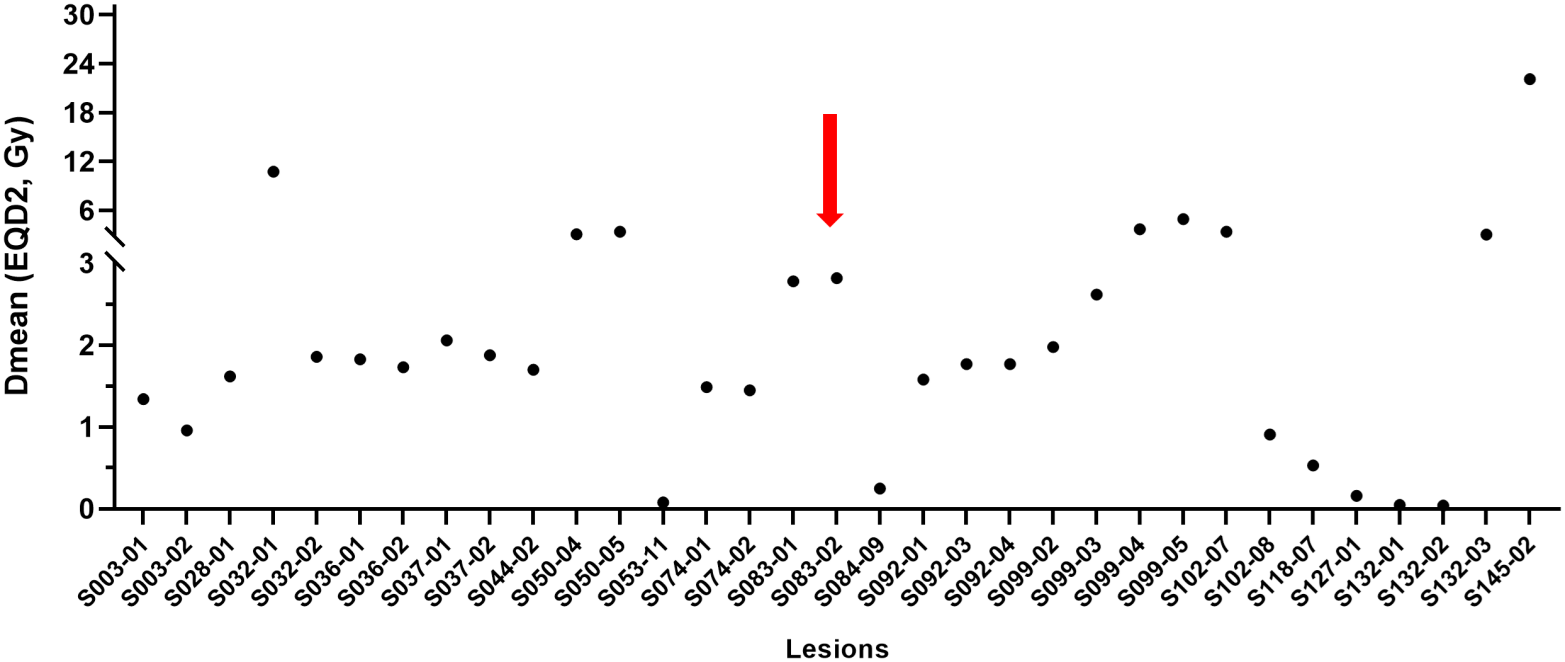
Mean dose profiles of metastatic non-targeted lesions inside the 0.5 Gy isodose line. Per-lesion analysis. The red arrow indicates a lesion showing the abscopal effect. Dmean, mean dose; EQD2, equivalent dose in 2-Gy fractions (α/β = 10).

Regarding each non-targeted metastatic lesion, including the primary breast, the AE was frequently seen in lymph nodes (n = 22, 59.5%) and lesions outside the simulation CT (n = 25, 67.6%; **Table S2**). The majority of the mean dose of 33 lesions inside the 0.5 Gy isodose line was under 3 Gy, and only one (3.0%) lesion had the AE (**Figure 4**).

**Figure 5:**
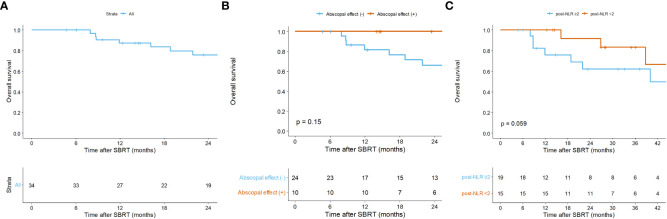
Kaplan-Meier curves of overall survival **(A)** of all patients, and according to **(B)** the abscopal effect and **(C)** the post-treatment neutrophil-to-lymphocyte ratio. Per-patient analysis. Post-NLR, post-treatment neutrophil-to-lymphocyte ratio; SBRT, stereotactic body radiotherapy.

Overall, in the per-treatment analysis, the 1-year PFS was 14.9% (95% CI = 6.8 – 32.6), and the median PFS was 5.5 months (95% CI = 3.5 – 8.5; **Figure 2B**). Though not statistically significant, the AE (+) group tended to have longer PFS than the AE (-) group (median PFS, 8.6 vs. 4.0 months, p = 0.160; **Figure 2C**). The patient-specific OS curves are shown in **Figure 5**. The OS rates at one and two years were 87.4% (95% CI = 76.6 – 99.7) and 76.0% (95% CI = 61.8 – 93.4), respectively (**Figure 5A**). The median OS was not reached. OS also showed a trend toward improvement in the AE (+) group (median OS, not reached vs. 40 months, p = 0.150; **Figure 5B**). Patients with a low post-treatment NLR (< 2) had a slightly superior OS compared to those with a high NLR (median OS, not reached vs. 40 months, p = 0.059; **Figure 5C**). Univariate analysis did not identify any prognostic factors that significantly impacted PFS and OS (**Table S3**).

## 4 Discussion

In our study, one-fourth of the patients with various metastatic lesions from breast cancer who underwent SBRT using CyberKnife developed the AE after approximately two months following SBRT. The PFR of these lesions was 70.0% at one year. To the best of our knowledge, this was the first study to find that not changing systemic treatment after SBRT was significantly linked to an increase in the AE. The post-treatment NLR had a marginal impact on the AE, and neither the dose nor volume of SBRT was related to the incidence of AE. Although we failed to find statistical significance, patients with the AE had longer median PFS and OS, suggesting a potential link between the AE and survival outcomes.

A phase II MD Anderson Cancer Center trial reported that the non-irradiated lesion-specific response of the high-dose radiotherapy and high-dose radiotherapy+LDRT group was 11% and 23%, respectively, with no significant difference noted (9). In our study, the doses to the non-targeted lesions inside the 0.5 Gy isodose line were lower than those in the phase II trial. Consequently, the lesions outside the SBRT targets in our patients can be considered non-irradiated lesions, leading to similar response rates. Notably, unlike the phase II trial, our patients did not receive immunotherapy, suggesting that SBRT alone played a role in the attainment of the AE. Rather, protecting the PIM from radiation-induced damage may be more pivotal because SBRT-PATHY itself exhibited a 45% incidence of the AE (10). Unfortunately, the PTV volume in our study was small, with a median of 40.81 cc, and the potentially affected PIM may have been relatively small as well, which could have contributed to the observed outcomes.

One novel finding of this study was that whether or not systemic treatment was changed after SBRT was an important factor in the development of the AE. More AE occurred when the systemic treatment regimen was not changed. The choice to change systemic treatment following SBRT was dependent on the physician, but no differences were observed in the palliative regimen lines and disease status before SBRT. Ultimately, the change in systemic treatment did not demonstrate any association with PFS and OS. In the phase II MD Anderson Cancer Center trial, 60 (95%) of 63 patients continued with the same treatment regimen after experiencing progression on immunotherapy (9).

We hypothesized that the change in systemic treatment affected both the tumor cells and the immune cells. This is based on the understanding that SBRT, in comparison to conventional RT, is recognized for its minimal impact on lymphocyte counts (11, 12). The changes in the numbers of WBCs, lymphocytes, and neutrophils in the blood after SBRT were analyzed. And the magnitude of the decrease was greater in neutrophils only in patients who switched systemic treatment compared to those who did not, whereas the difference in the decrease in WBCs and lymphocytes was not significant between the two groups. It is well known that neutrophils are the most decreased immune cells after chemotherapy (13). Neutrophils are attracted to the tumor microenvironment through chemokine receptors, CXCR1 and CXCR2, which are expressed at high levels on the surface of neutrophils (14). Neutrophils have antitumor activity via an oxidative burst of reactive oxygen species (15, 16). These neutrophils also mature into antigen-presenting cell-like neutrophils and activate antitumor adaptive immunity, such as the T-cell response (17). Golden et al. reported that granulocyte-macrophage colony-stimulating factor combined with RT produced an objective AE (18). Therefore, a large decrease in neutrophils may reduce the systemic immune response to the tumor.

Of the lesions that were reduced by the AE, 30% showed progression one year after AE. The longer median PFS and OS in patients in the AE (+) group suggested a potential link between the AE and survival outcomes. A systematic review of metastatic lung cancer revealed that the AE had the potential to delay the progression of the disease, but it had no clinical benefit to OS (7). The clinical benefits of the AE in terms of PFS and OS warrant further investigations into how to enhance the AE in patients with metastatic cancer.

It is important to acknowledge the limitations of this study. First, this was a retrospective cohort study, which had inherent flaws, including selection bias. The sample size of this study was relatively small, which may constrain the statistical power. Due to the lack of tissue samples, we only used immune cell counts from peripheral blood, but it is unknown whether this would correlate with changes in immune cells in the actual tumor tissue.

We have initiated a phase II prospective cohort study, the Korean Radiation Oncology Group 22-11 (NCT05733156), to enhance the AE by adding LDRT to SBRT in patients with solid tumors. This trial is recruiting 52 participants who will receive 3 fractions of SBRT and LDRT simultaneously. This study does not consider previous immunotherapy as an inclusion criterion. The primary endpoint is the AE rate of LDRT lesions three months after the completion of RT.

In conclusion, our research found that one in every four patients with metastatic breast cancer experienced the AE after SBRT without immunotherapy. Lesions with the AE had a 1-year PFR of 70.0%, which somewhat influenced the overall treatment outcomes. The AE was not affected by the dose and volume of SBRT, but we did find that not changing systemic treatment after SBRT was significantly associated with the AE. Additionally, a low post-treatment NLR may indicate a positive response to SBRT non-targeted lesions. Further validation is needed.

## Data availability statement

The raw data supporting the conclusions of this article will be made available by the authors, without undue reservation.

## Ethics statement

The studies involving humans were approved by the Institutional Review Board at Soonchunhyang University Seoul Hospital. The studies were conducted in accordance with the local legislation and institutional requirements. The ethics committee/institutional review board waived the requirement of written informed consent for participation from the participants or the participants’ legal guardians/next of kin because of the retrospective review and minimal risk to patients.

## Author contributions

AC contributed to the conception and design of the study. JK organized the database, performed the statistical analyses, and wrote the first draft of the manuscript. All authors contributed to the manuscript revision and read and approved the final manuscript.
