# Supplementary material for: Abscopal effect in metastatic breast cancer treated with stereotactic body radiotherapy in the absence of immunotherapy
Source: Front Oncol. 2023 Oct 6;13:1243053. doi: 10.3389/fonc.2023.1243053 (PMC10587686; doi:10.3389/fonc.2023.1243053)
Supplement: Supplementary file 1 [file Table_1.docx]

Table S1. Abscopal effect according to change in the systemic treatment after stereotactic body radiotherapy^*^

| **Systemic treatment** | **Abscopal effect (-),**  **N=30** | **Abscopal effect (+),**  **N=10** | **p-value** |
| --- | --- | --- | --- |
| No change | 7 (23.3) | 7 (70.0) | 0.027 |
| Change | 22 (73.3) | 3 (30.0) |  |
| Stop | 1 (3.3) | 0 (0.0) |  |

Values are described as N (%).

^*^Per-treatment analysis.
