# Supplementary material for: Abscopal effect in metastatic breast cancer treated with stereotactic body radiotherapy in the absence of immunotherapy
Source: Front Oncol. 2023 Oct 6;13:1243053. doi: 10.3389/fonc.2023.1243053 (PMC10587686; doi:10.3389/fonc.2023.1243053)
Supplement: Supplementary file 2 [file Table_2.docx]

Table S2. Lesion-specific abscopal effect^*^

| **Characteristics** | **Abscopal effect (-),**  **N=112** | **Abscopal effect (+),**  **N=37** | **p-value** |
| --- | --- | --- | --- |
| Lesion site |  |  | 0.225 |
| Lymph node | 49 (43.8) | 22 (59.5) |  |
| Lung | 26 (23.2) | 6 (16.2) |  |
| Breast | 17 (15.2) | 7 (18.9) |  |
| Liver | 9 (8.0) | 0 (0.0) |  |
| Pleura | 5 (4.5) | 0 (0.0) |  |
| Others | 6 (5.4) | 2 (5.4) |  |
| Lesion location in simulation CT |  |  | <0.001 |
| Not covered | 37 (33.0) | 25 (67.6) |  |
| < 0.5 Gy isodose line | 43 (38.4) | 11 (29.7) |  |
| ≥ 0.5 Gy isodose line | 32 (28.6) | 1 (2.7) |  |

Values are described as N (%).

^*^Per-lesion analysis.

CT, computed tomography.
