# Supplementary material for: Abscopal effect in metastatic breast cancer treated with stereotactic body radiotherapy in the absence of immunotherapy
Source: Front Oncol. 2023 Oct 6;13:1243053. doi: 10.3389/fonc.2023.1243053 (PMC10587686; doi:10.3389/fonc.2023.1243053)
Supplement: Supplementary file 3 [file Table_3.docx]

Table S3. Analysis of the prognostic factors for progression-free^*^ and overall survival^†^

|  | **Progression-free survival** | | **Overall survival** | |
| --- | --- | --- | --- | --- |
| **Variables** | **Hazard ratio (95% CI)** | **P-value** | **Hazard ratio (95% CI)** | **P-value** |
| HR+/HER2- subtype | 0.664 (0.338-1.307) | 0.236 | 0.903 (0.276-2.956) | 0.865 |
| Primary breast lesion present | 0.583 (0.837-3.517) | 0.140 | 0.966 (0.323-2.889) | 0.950 |
| Chemotherapy before SBRT | 1.603 (0.796-3.229) | 0.187 | 1.341 (0.400-4.493) | 0.635 |
| Targeted therapy before SBRT | 0.962 (0.483-1.917) | 0.913 | 2.400 (0.664-8.669) | 0.182 |
| Hormone therapy before SBRT | 0.590 (0.280-1.243) | 0.165 | 0.603 (0.163-2.237) | 0.450 |
| No change in systemic therapy after SBRT | 0.944 (0.472-1.889) | 0.872 | 0.947 (0.190-4.721) | 0.947 |
| Interval between SBRT and systemic therapy (incremental) | 1.002 (0.997-1.008) | 0.467 | 1.000 (0.992-1.007) | 0.962 |
| Post-treatment NLR < 2 | 0.691 (0.358-1.333) | 0.270 | 0.308 (0.084-1.124) | 0.075 |
| SBRT EQD2 > 42.75 Gy | 1.140 (0.579-2.246) | 0.704 | 1.323 (0.441-3.967) | 0.617 |
| PTV > 40 cc | 0.930 (0.482-1.796) | 0.829 | 2.099 (0.644-6.839) | 0.218 |
| Abscopal effect observed | 0.571 (0.257-1.266) | 0.167 | 0.243 (0.031-1.915) | 0.179 |

^*^Per-treatment analysis.

^†^Per-patient analysis.

CI, confidence interval; EQD2, equivalent dose in 2-Gy fractions (α/β=10); HER2, human epidermal growth factor receptor 2; HR, hormone receptor; NLR, neutrophil-to-lymphocyte ratio; PTV, planning target volume; SBRT, stereotactic body radiotherapy.
